# Fused Right Supernumerary Kidney: A Case Report

**DOI:** 10.31729/jnma.4563

**Published:** 2019-10-31

**Authors:** Sagun Manandhar, Ashish Khanal

**Affiliations:** 1 Department of Radiology and Imaging, Patan Academy of Health Sciences, Lalitpur, Nepal

**Keywords:** *congenital anomaly*, *CT urography*, *fused*, *supernumerary kidney*

## Abstract

Supernumerary kidney is a rare clinical entity with fused supernumerary kidney being even rarer. Caudally located fused right supernumerary kidney with multiple nephrolithiasis was diagnosed in a 69-years-old lady by Computed Tomography Urography. A separate renal artery arising from the abdominal aorta as well as separate renal vein draining into the inferior vena cava was present along with right sided bifid collecting system. Embryological basis of origin of supernumerary kidney, its diagnosis, clinical significance and management are discussed.

## INTRODUCTION

Supernumerary kidney refers to the presence of an extra kidney in addition to two independent kidneys, which is a rare congenital anomaly. True incidence of supernumerary kidney cannot be stated due to its rarity, and fused supernumerary kidney is even rarer. A supernumerary kidney has its own collecting system, vascular supply and distinct encapsulated parenchyma.^1^ Here, a rare case of fused right supernumerary kidney in a 69 years old elderly female is reported.

## CASE REPORT

Sixty nine years old lady presented to outpatient department with complaint of on and off dull aching pain over right upper abdomen for past two months and increasing in severity for the past two weeks. She did not have a history of fever, vomiting, increased frequency of micturition, burning micturition or blood in urine. Per abdomen examination was unremarkable. Routine hematological and biochemical investigations were within normal limits. Urinalysis showed three to five red blood cells per high power field. Ultrasonography showed two calculi in lower pole calyx of right kidney with focal caliectasis. She underwent Computed Tomography (CT) urography with contrast administration which showed two fused right kidneys and a single left kidney.

**Figure 1:**
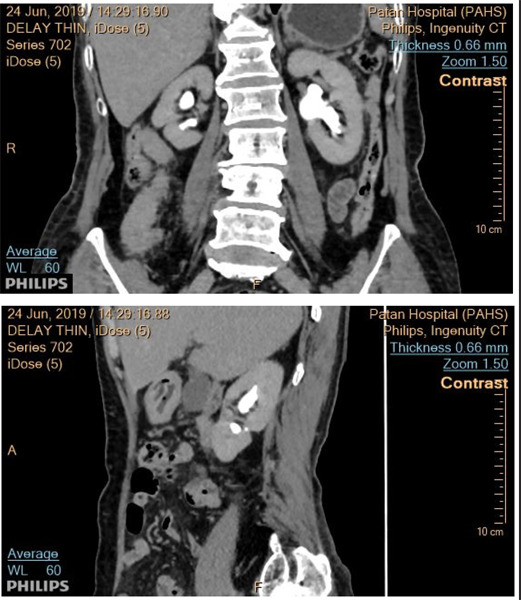
Coronal (A) and right sagittal (B) reconstructed CT urography images in excretory phase showing caudally located fused right supernumerary kidney (arrow) and normal left kidney.

**Figure 2:**
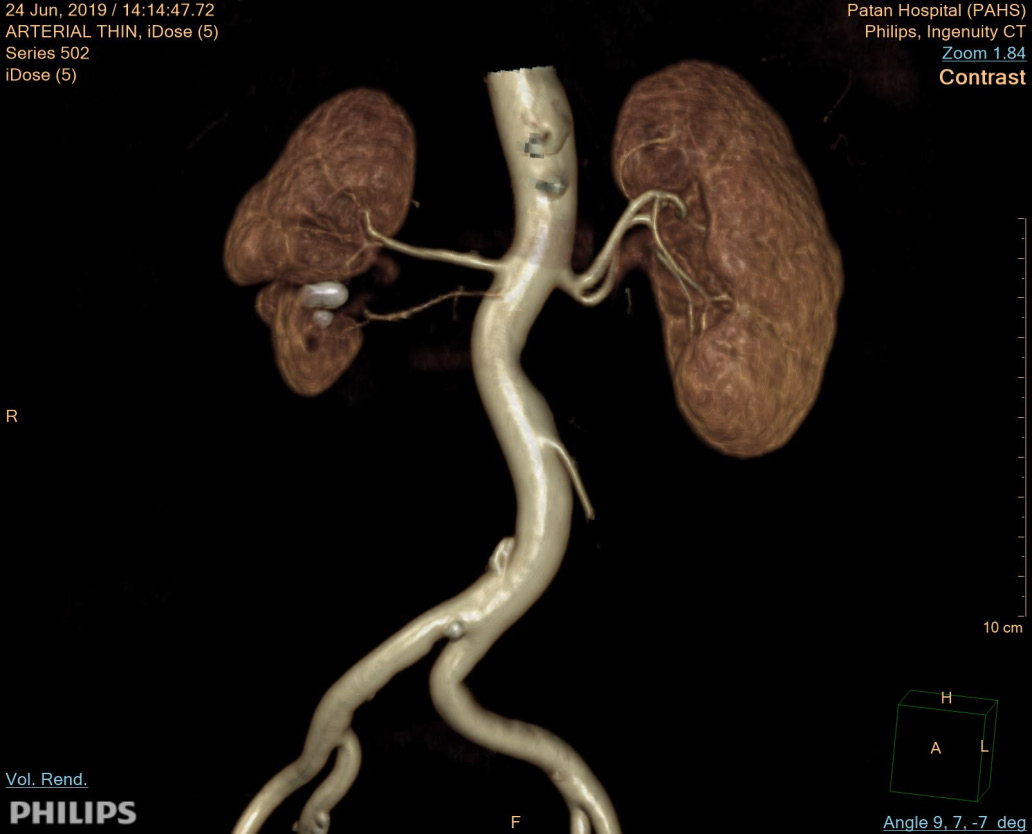
3D reconstruction of CT urography image in arterial phase showed a separate arterial supply to right supernumerary kidney from abdominal aorta and early segmental branching of left renal artery. Two large calculi in the fused right supernumerary kidney were also appreciated.

**Figure 3:**
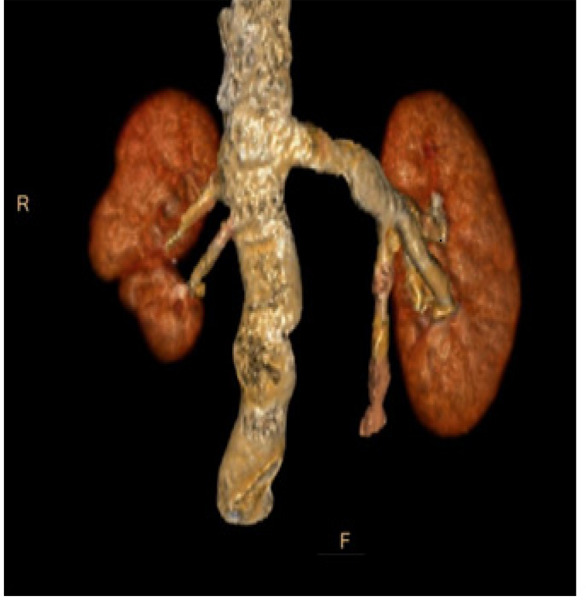
3D reconstruction of CT urography image in venous phase showed a separate vein from right supernumerary kidney draining into the inferior vena cava.

**Figure 4:**
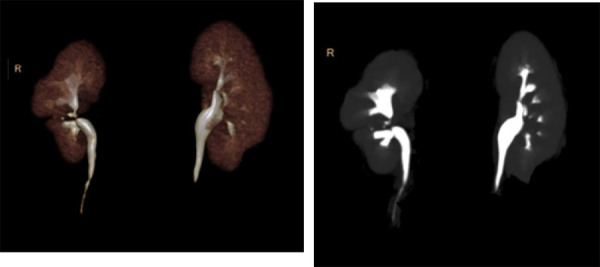
3D reconstruction (A) and maximum intensity projection (B) of CT urography image in excretory phase showed a separately draining calyceal system of caudal right supernumerary kidney uniting with calyceal system of cranial right kidney at the extrarenal pelvis and having a single common right ureter.

Cranially located right kidney measured 6.4 cm in craniocaudal and 3 cm in mediolateral dimensions, and had normal arterial supply and vascular drainage. Caudally located kidney on the right side was mildly deformed and smaller in size measuring 3.7 cm in craniocaudal and 2.3 cm in mediolateral dimensions (Figure 1). It had a separate renal artery arising from the abdominal aorta and passing anterior to the inferior vena cava (Figure 2). A separate renal vein draining into the inferior vena cava was also present (Figure 3). Right sided collecting system appeared as a bifid collecting system. Prompt excretion of contrast was seen from the larger cranial right kidney whereas deformed caudal right kidney showed delayed excretion. Both kidneys had a separately draining calyceal system uniting at the extrarenal right pelvis and had a single common right ureter (Figure 4). There were two large calculi in the caudal right kidney.

Left kidney was normal in outline and measured 8 cm in craniocaudal and 4.2cm in mediolateral dimensions. There was an early segmental branching of the left main renal artery, 4mm from its origin from abdominal aorta. There was no variation in venous drainage and collecting system of the left kidney, except for extrarenal pelvis. Bilateral ureters and urinary bladder had normal findings in the CT urography.

## DISCUSSION

A supernumerary kidney is uncommon but normally occurs on the left. It is usually located caudal to the normal kidney^1^ but may be found in the iliac or sacral region.^2^ Embryologically, supernumerary kidneys develop from abnormal division of the nephrogenic cord into two metanephric blastemas with completely or partially duplicated ureteral bud.^3^ An important entity from which supernumerary kidney should be distinguished is a frequently identified duplex kidney which has two pelvicalyceal system. Supernumerary kidney however has a separate arterial supply, venous drainage, collecting system and is either totally separate from the normal kidney or connected to it by loose areolar tissue.^4-7^

Pathologies of the urinary tract have been reported in patients with supernumerary kidneys with nephrolithiasis and hydronephrosis being common.^3^ In our case as well, large calculi were noted in the fused right supernumerary kidney. Various congenital anomalies like coarctation of aorta, horseshoe kidneys, ectopic ureteral opening, duplication of penis or female urethra and vaginal atresia are also found to be associated with supernumerary kidney.^8^ In our case however, none of these anomalies were present.

Supernumerary kidney is usually asymptomatic and discovered as an incidental finding. However patients with supernumerary kidney may present with a palpable abdominal mass, pain, fever or hypertension.^2^ Complications like pyelonephritis, pyonephrosis, hydronephrosis and malignant changes (clear cell carcinoma, Wilms’ tumor) have also been reported.

Nephrectomy is warranted in non-functional kidney status or presence of renal disease, otherwise regular follow up is suggested.^3^ Modality and time interval of follow up has not been well established. Surgical intervention in case of supernumerary kidney is challenging due to its peculiar vascular supply and thus correct diagnosis prior to surgery is of utmost important. CT urography sufficed for diagnosis in our case. Intravenous pyelography, ultrasonography, nuclear scintigraphy and MRI with MR angiography can also be alternatives for diagnosing supernumerary kidney.

Consent: JNMA Case Report Consent Form was signed by the patient and the original article is attached with the patient’s chart.

## Conflict of Interest:

None.
